# Lichen Planus Pigmentosus Inversus: A Case Report of a Man Presenting With a Pigmented Lichenoid Axillary Inverse Dermatosis (PLAID)

**DOI:** 10.7759/cureus.56995

**Published:** 2024-03-26

**Authors:** Philip R Cohen, Christof P Erickson, Antoanella Calame

**Affiliations:** 1 Dermatology, University of California Davis Health, Sacramento, USA; 2 Dermatology, Touro University California College of Osteopathic Medicine, Vallejo, USA; 3 Dermatology, Compass Dermatopathology, San Diego, USA; 4 Dermatology/Dermatopathology, Compass Dermatopathology, San Diego, USA; 5 Dermatology, Scripps Memorial Hospital, La Jolla, USA

**Keywords:** tacrolimus, planus, plaid, pigmentosus, lichenoid, lichen, inversus, intertriginous, hyperpigmented, axilla

## Abstract

Lichen planus pigmentosus is an uncommon subtype of lichen planus and lichen planus pigmentosus inversus is a rare variant of lichen planus pigmentosus. Lichen planus pigmentosus inversus typically presents as hyperpigmented patches or plaques, particularly in the intertriginous areas such as the axillae, the groin and inguinal folds, and in the submammary region. In some patients with lichen planus pigmentosus inversus, the condition can present as a pigmented lichenoid axillary inverse dermatosis (PLAID) when the lesions are in the axillae. A 49-year-old Hispanic man who had hyperlipidemia and diabetes mellitus developed lichen planus pigmentosus inversus and presented with a PLAID. Skin biopsies established the diagnosis of lichen planus pigmentosus inversus. The clinical differential diagnosis of lichen planus pigmentosus inversus includes inherited disorders, primary cutaneous dermatoses, acquired dyschromias, and reactions to topical or systemic medications. Friction in intertriginous areas has been related to the development of lichen planus pigmentosus inversus. Factors that can precipitate lichen planus pigmentosus inversus include not only topical exposure to almond oil, amala oil, cold and cosmetic creams, henna, and paraphenyldiamine but also either topical contact or consumption of mustard oil and nickel. Lichen planus pigmentosus inversus can be associated with autoimmune conditions (hypothyroidism), endocrinopathies (diabetes mellitus), and hyperlipidemia. The dyschromia found in patients with lichen planus pigmentosus inversus is frequently refractory to treatment. Initial management includes removal of potential disease triggers such as eliminating tight clothing to stop friction with the adjacent skin. Topical corticosteroids do not result in improvement; however, topical calcineurin inhibitors such as tacrolimus have been reported to be efficacious. In conclusion, inverse lichen planus and lichen planus pigmentosus inversus can present with a PLAID; whereas topical corticosteroids may be helpful to resolve inverse lichen planus lesions, topical tacrolimus may be useful to improve the dyschromia in lichen planus pigmentosus inversus.

## Introduction

Lichen planus is a papulosquamous skin condition. It can have manifestations that not only affect the skin and mucous membranes but also the hair and nails. Lichen planus has several clinical variants [1,2].

Inverse lichen planus is a less frequently occurring variant of the condition. It is characterized by lesions that appear in intertriginous areas. Lesions may be present in the axilla, inframammary folds, and groin [1,2].

Lichen planus pigmentosus is an uncommon subtype of lichen planus; albeit less commonly, classical lichen planus lesions have been observed in patients with lichen planus pigmentosus [3-9]. Lichen planus pigmentosus usually appears on sun-exposed body sites [3,4,7-10]. Lichen planus pigmentosus inversus is a rare variant of lichen planus pigmentosus; similar to inverse lichen planus, lichen planus pigmentosus inversus occurs in intertriginous locations [4,11-20].

Both inverse lichen planus and lichen planus pigmentosus inversus can present as a pigmented lichenoid axillary inverse dermatosis (PLAID) [1,2,10,12,20]. A man with hyperlipidemia and diabetes mellitus who developed a PLAID is described. The diagnosis of the man’s cutaneous dermatosis, lichen planus pigmentosus inversus, was established based on the correlation of an absence of skin disease-related symptoms, the morphology of the cutaneous lesions exclusively restricted to his axilla, and the pathologic changes observed on the lesional skin biopsies.

Lichen planus pigmentosus inversus typically presents with nonpruritic intertriginous hyperpigmented patches or plaques that morphologically mimic inverse lichen planus. External mechanical irritation from friction or tightly fitting underclothes may predispose to the development of lichen planus pigmentosus inversus. The dyschromia resulting from lichen planus pigmentosus inversus can be an issue of cosmetic concern for affected individuals; however, in contrast to inverse lichen planus, the typically asymptomatic hyperpigmentation of lichen planus pigmentosus inversus is often refractory to therapeutic interventions [3-9,11-20].

## Case presentation

A 49-year-old Hispanic man, Fitzpatrick skin type V, presented for the evaluation of newly acquired discoloration of both of his armpits. His past medical history was significant for sleep apnea; he had used a continuous positive air pressure machine at night for two years. He also had hyperlipidemia which was treated with 20 mg of atorvastatin calcium daily, and type II diabetes mellitus of 10 years duration which was managed with 1000 mg of metformin hydrochloride twice daily and 5 mg of linagliptin once daily. His medical history was unremarkable for hepatitis and hypothyroidism.

Both of his armpits have had skin tags that had been present for several years. Two months ago, he noted new asymptomatic red lesions that subsequently became surrounded by dark areas in both armpits. Eventually, the lesions became completely dark.

**Figure 1:**
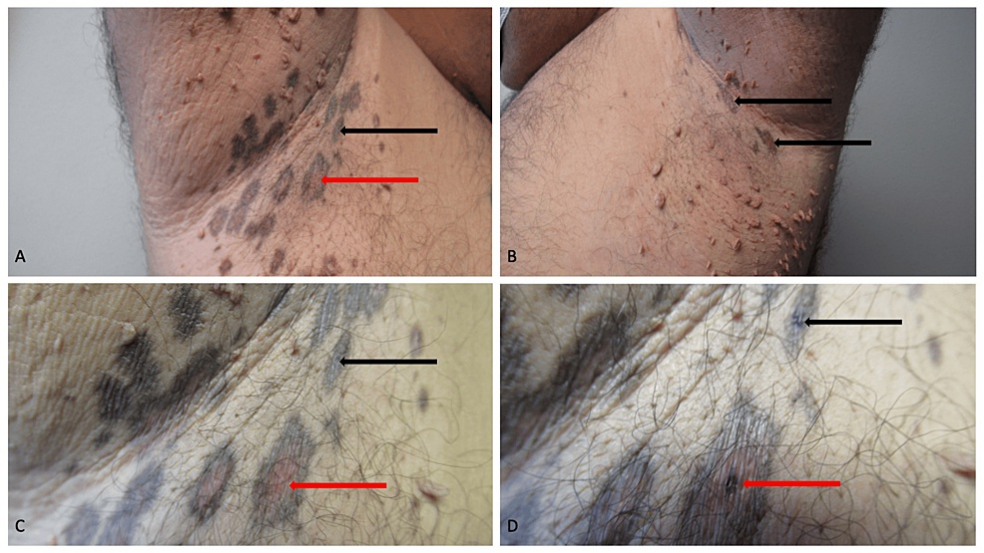
Clinical presentation of lichen planus pigmentosus inversus in a 49-year-old Hispanic man Distant (A and B) and closer (C and D) views of the right axilla (A, C, and D) and the left axilla (B) demonstrate lesions of lichen planus pigmentosus inversus. In addition to numerous skin tags, both axillae show scaly hyperpigmented plaques and patches (black arrows). The right axilla also shows central red areas in some of the dark lesions (red arrows). The purple spots (D) show the location of the biopsies or the red area (red arrow) and dark area (black arrow).

Cutaneous examination of the axillae was remarkable for scaly plaques (Figure 1). Some of the plaques in the left axilla had central red areas with surrounding hyperpigmentation; however, in the right axilla, only scaly hyperpigmented brown plaques were noted. Both axillae had numerous soft pedunculated tag-like skin lesions.

There were no other areas of red or hyperpigmented plaques on the body. Evaluation of his hair, nails, and mucous membranes were unremarkable. There was neither alopecia, nor nail dystrophy, nor lesion involving the oral, genital, or perianal areas.

Two skin biopsies were performed using the punch technique (Figure 1). The central red area was biopsied. In addition, a confluent hyperpigmented plaque was biopsied.

**Figure 2:**
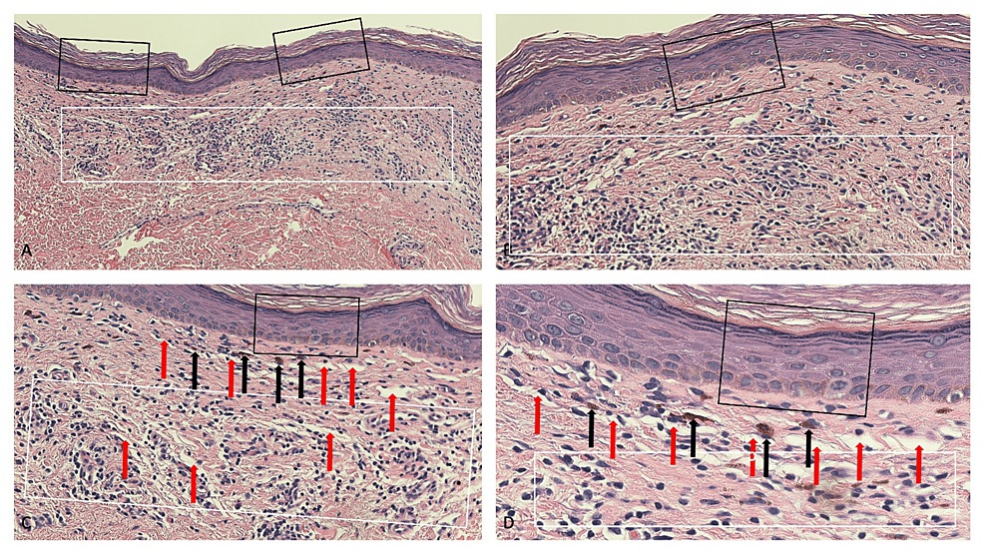
Pathologic changes observed in the red area of lichen planus pigmentosus inversus lesion Low-magnification (A) and higher-magnification (B, C, and D) views of the pathologic features of lichen planus pigmentosus inversus of the red area within a plaque. Microscopic examination shows a flattening of the epidermal rete ridges with atrophy of the epidermis; there is also sparse basket weave hyperkeratosis which clinically correlates with the slight scale that was noted (black rectangles). In the upper dermis, there is a lymphocytic inflammation that is band-like in distribution (white rectangle). A few pigmented melanophages are seen in the upper dermis (black arrows), and several telangiectatic blood vessels (red arrows) are also prominent (hematoxylin and eosin: A, x10; B, x20; C, x20; D, x40).

Microscopic examination of the red area within a plaque showed atrophy of the epidermis with flattening of the epidermal rete ridges; sparse orthokeratosis, which correlated clinically with the scaling observed, was noted overlying the epidermis (Figure 2). A band-like infiltrate of lymphocytes was present in the upper dermis. Numerous telangiectatic blood vessels and a small number of pigmented melanophages were noted in the dermis.

**Figure 3:**
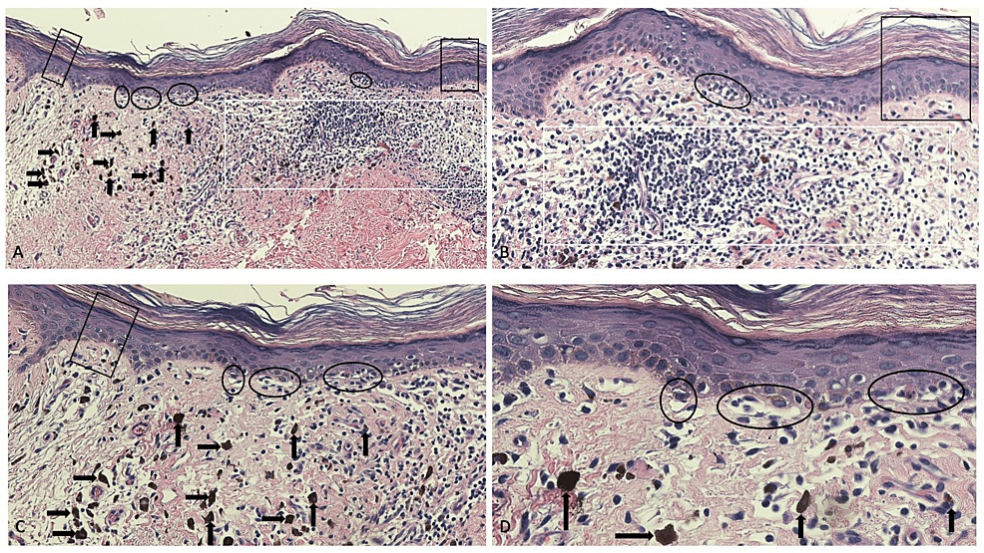
Microscopic examination of hyperpigmented lichen planus pigmentosus inversus plaque Low-magnification (A) and higher-magnification (B, C, and D) views of the microscopic findings of the dark plaque of lichen planus pigmentosus inversus show sparse orthokeratosis; there is atrophy of the underlying epidermis with flattening of the rete ridges (black rectangles). In the basal layer of the epidermis, liquefaction degeneration (also known as vacuolar alteration) is focally present (black ovals). A lichenoid infiltrate of lymphocytes is noted in the upper dermis (white rectangle). In the upper dermis, there is edema, and numerous melanophages (black arrows) can be observed (hematoxylin and eosin: A, x10; B, x20; C, x20; D, x40).

Microscopic examination of the dark plaque showed some similar features to those observed in the red plaque (Figure 3). There was sparse orthokeratosis with underlying epidermal atrophy; focally, there was a vacuolar alteration of the cells in the basal layer of the epidermis. In the upper dermis, there was a lichenoid infiltrate of chronic inflammatory cells. Edema and numerous melanophages were present in the upper dermis.

The correlation of the clinical presentation, lesion morphology, and pathology findings established the diagnosis of lichen planus pigmentosus inversus. The red plaques were spontaneously becoming hyperpigmented; since the lesions were not symptomatic, the patient decided to monitor them clinically. He also elected to observe the pedunculated acrochordons that were concurrently present in his axilla.

## Discussion

Acquired dyschromia can be associated with either systemic conditions, mucocutaneous disorders, or drug reactions. The distribution of the skin darkening may be related to the etiology of the condition. The affected body locations can range from being diffuse, or areas that are sun-exposed, or less commonly restricted to intertriginous sites [1-20].

Intertriginous locations include the abdominal folds, antecubital fossa, axilla, groin, inguinal folds, popliteal fossa, and submammary region. Occasionally, dyschromia is restricted to the axilla. When the axillary hyperpigmentation is the result of a lichenoid lymphocytic inflammatory process that is presenting in a distribution that is inverse and opposite to the body areas typically affected by the condition, it can be referred to as a pigmented lichenoid axillary inverse dermatosis or PLAID [1-20].

Inverse lichen planus and lichen planus pigmentosus inversus are two variants of lichen planus that can be localized to the axilla. Lichen planus often appears in flexural areas; however, when intertriginous sites predominate, the condition is designated as the less common subtype of inverse lichen planus. Lichen planus pigmentosus is an infrequent variant of lichen planus that is typically localized to sun-exposed areas; rarely, this subtype of lichen planus concurrently or only involves intertriginous locations and is described as lichen planus pigmentosus inversus [1-20].

The term lichen pigmentosus initially appeared in the Japanese literature. Earlier researchers speculated that the same condition may have been reported in the French literature under a different nomenclature: "lichens atypiques ou invisible pigmentogenes." Subsequently, in 2001, Pock et al. introduced the variant of lichen planus pigmentosus localized to the intertriginous sites: lichen planus pigmentosus inversus [4,12].

Lichen planus pigmentosus inversus was initially described in seven Caucasian individuals [12]. However, larger retrospective studies of lichen planus pigmentosus and lichen planus pigmentosus inversus patients have observed that the individuals typically have darker skin tones, such as Fitzpatrick skin types III to VI [4]. There is a slight prevalence of women in their third or fourth decades of life as compared to men with lichen planus pigmentosus [8,9].

The hyperpigmentation that occurs in patients with lichen planus pigmentosus is often in sun-exposed locations. The patterns of pigment can be diffuse, blotchy, reticulate, or perifollicular. In addition, the distribution of pigmentation can be symmetrical, segmental, linear, zosteriform, or blaschkoid [5,7,8].

In contrast to classic lichen planus, the lesions of lichen planus pigmentosus and lichen planus pigmentosus inversus are frequently asymptomatic [18]. Occasionally, the cutaneous lesions have been described as itching or burning. In Pock et al.’s original description of lichen planus pigmentosus inversus, three of the patients had no itching, and the remaining four individuals only had slight pruritus [12].

In a study of 100 lichen planus pigmentosus patients, 30 patients were asymptomatic, and 41 patients had itching [5]. In another study of 124 lichen planus pigmentosus patients, 62% (77 patients) were asymptomatic, 31% (39 patients) had mild to moderate itching, and 6.5% (eight patients) had a burning sensation [8]. When itching is present in lichen planus pigmentosus patients, it has been considered as a marker of both disease activity and progression [5].

Lichen planus pigmentosus can occur in the absence of classical lichen planus. In a retrospective study of 100 lichen planus pigmentosus patients, none of the patients had concomitant lichen planus [5]. However, in a retrospective study of 124 lichen planus pigmentosus patients, 15% (19 patients) also had coincidental lichen planus [8]. 

In lichen planus pigmentosus inversus, the designation inverse has been adopted. The term describes the distribution of the pigmented lesions in lichen planus pigmentosus inversus that are anatomically distributed in the skin folds that is opposite to that observed in lichen planus pigmentosus in which the lesions appear on sun-exposed skin [18]. Importantly, lichen planus pigmentosus inversus can occur as a component of lichen planus pigmentosus with skin lesions also being present in intertriginous areas [3-5,7].

However, patients with lichen planus pigmentosus inversus may only demonstrate cutaneous lesions restricted to the intertriginous areas. Indeed, in Pock et al’s. initial description of the condition, four of the seven patients only had intertriginous lesions that were restricted to only the axilla (one patient), the axilla and groin (two patients), or the axilla, groin, and submammary area (one patient) [12]. In another retrospective study of 100 lichen planus pigmentosus patients, 13% of the patients had lesions only restricted to their intertriginous areas or concurrently present with non-intertriginous lichen planus pigmentosus lesions [5].

The clinical morphology of a lichen planus pigmentosus inversus lesion is similar to that of lichen planus pigmentosus; however, the essential difference between these variants of lichen planus is the location of the lesions. Both lichen planus pigmentosus inversus and lichen planus pigmentosus are often macular patches of dyschromia; occasionally, similar to the reported patient, they can be slightly scaly. In contrast to lichen planus, Wickham striae are usually absent in lichen planus pigmentosus [3].

Hyperpigmentation in lichen planus pigmentosus inversus and lichen planus pigmentosus ranges from blue to violaceous to dark brown and brownish black to gray [3,6,7,11,12,17]. The lesions of the reported patient had two different clinical presentations. Some of the lesions in the right axilla had central erythema with surrounding hyperpigmentation; we speculate that the redness clinically observed in the erythematous area may be secondary to the increased number telangiectatic vessels and paucity of melanophages. In contrast, numerous melanophages in the upper dermis were noted in the reported patient’s hyperpigmented lesion.

Lichen planus pigmentosus does not commonly have oral lesions [7]. In a retrospective study of 30 lichen planus pigmentosus patients, 16.7% (five patients) had oral involvement [9]. In another retrospective study of 100 lichen planus pigmentosus patients, 8% (eight patients) had disease-related oral lesions; the bilateral buccal mucosa showed violaceous pigmentation [5]. In a third study of 124 lichen planus pigmentosus patients, only 3% (four patients) had oral lesions [8]. 

Nail involvement in lichen planus pigmentosus has been described in individual case reports and has also been observed in some of the retrospective studies of lichen planus pigmentosus patients. A 59-year-old woman presented with lichen planus pigmentosus, nail lichen planus, and frontal fibrosing alopecia [6]. In a study of 30 lichen planus pigmentosus patients, 23.3% (seven patients) had nail involvement [9]. In a larger study of 124 lichen planus pigmentosus patients, only 14% (16 patients) had disease-related nail dystrophy: 12 patients had longitudinal ridging, and four patients had longitudinal melanonychia [5].

An association has been observed of lichen planus pigmentosus and frontal fibrosing alopecia [6,17]. It has been speculated that the pathogenesis of lichen planus pigmentosus and frontal fibrosing alopecia may be related since both conditions can have similar pathologic findings [6]. However, disease-related scalp lesions and a scarring lichenoid alopecia are not typically present in patients with lichen planus pigmentosus inversus [1,20].

Lichen planus pigmentosus inversus is not usually associated with mucosal lesions or nail lesions [1,20]. However, nail involvement with lichen planus pigmentosus inversus has recently been described. A 52-year-old man developed not only a pruritic dermatosis in the axilla and groin but also nail changes; his biopsy-confirmed skin lesions of lichen planus pigmentosus inversus appeared one week, and his disease-related nail changes presented three weeks, after his third dose of Coronavirus disease 2019 (COVID-19) vaccine [17].

The clinical differential diagnosis of lichen planus pigmentosus (Table 1) and lichen planus pigmentosus inversus (Table 2) includes inherited disorders, primary cutaneous dermatoses, acquired dsychromias, and reactions to topical or systemic medications [1,3-5,7,10-13,19]. Erythema dyschromicum perstans clinically presents similar to lichen planus pigmentosus. However, the early phase of erythema dyschromicum perstans can present with an erythematous border [5].

**Table 1:** Clinical differential diagnosis of lichen planus pigmentosus ^a^Some researchers consider ashy dermatosis and erythema dyschromicum perstans to be the same condition. ^b^Urticaria pigmentatosa is a subtype of mastocytosis. ^c^Nevus of Hori is also referred to as nevus fusco-caeruleus zygomaticus, acquired circumscribed dermal facial melanocytoses, and acquired bilateral nevus of Ota-like macules; it presents as bilateral blue-gray facial macules. ^d^Nevus of Ota, also known as ocular dermal melanosis, is usually unilateral and most commonly involves the ophthalmic (first division, V1) and the maxillary (second division, V2) of the trigeminal nerve; associated hyperpigmentation of the eye is present.

Condition	References
Actinic lichen planus	[7,9,16]
Ashy dermatosis^a^	[4,5,7,19]
Contact dermatitis (pigmented)	[4]
Dermal melanosis	[5]
Drug eruption (cutaneous)	[10]
Drug eruption (generalized fixed)	[3,4]
Drug eruption (hyperpigmentation)	[3,7]
Erythema dyschromicum perstans^a^	[1,3-5,7,20]
Figurate erythema	[1]
Friction-induced hyperpigmentation	[3]
Heavy metal reaction	[3]
Idiopathic eruptive macular pigmentation	[3,4]
Macular amyloidosis	[3,5]
Mastocytosis^b^	[7]
Melasma	[4]
Nevus of Hori^c^	[4]
Nevus of Ota^d^	[5]
Ochronosis	[4]
Post-inflammatory hyperpigmentation	[1,3,4,7]
Riehl’s melanosis	[4,10,19]
Systemic lupus erythematosus	[10]
Urticaria pigmentatosa^b^	[3]

**Table 2:** Clinical differential diagnosis of lichen planus pigmentosus inversus ^a^Some researchers consider ashy dermatosis and erythema dyschromicum perstans to be the same condition. ^b^These conditions often result in post-inflammatory hyperpigmentation of the affected areas. ^c^This includes reactions to friction to the area. ^d^The infectious agent in this condition is usually *Candida albicans*.

Condition	References
Acanthosis nigricans	[4,11,13]
Ashy dermatosis^a^	[11,19]
Axillary granular parakeratosis	[19]
Contact dermatitis^b^	[13,19]
Drug reactions	[5]
Erythema dyschromicum perstans^a^	[13]
Erythrasma	[4,11]
External stimuli-induced hyperpigmentation^c^	[11]
Figurate erythema	[19]
Fixed drug eruption	[11,13]
Intertrigo	[11]
Lichenoid toxic dermatitis	[11,19]
Occupational dermatoses^b^	[19]
Post-inflammatory hyperpigmentation	[11,13,19]

Indeed, some researchers have postulated that lichen planus pigmentosus and erythema dyschromicum perstans may be the same condition or represent disorders that are pathogenically related. However, a biopsy of the lesion may help to differentiate the conditions. Melanophages are typically located in the superficial dermis in lichen planus pigmentosus; in contrast, melanophages are usually located deeper in the dermis in erythema dyschromicum perstans [3-5].

Inverse lichen planus can be morphologically identical to lichen planus pigmentosus inversus. However, pruritus is more common in inverse lichen planus. Also, in contrast to lichen planus pigmentosus inversus, a biopsy of inverse lichen planus is more likely to demonstrate acanthosis, hypergranulosis, and extension of the rete ridges into the papillary dermis [3-5].

Pathologic changes observed in lesions of lichen planus pigmentosus and lichen planus pigmentosus inversus are the same. Vacuolar alteration of the epidermal basal layer, also referred as basal layer liquefaction or hydropic degeneration, is typically present in newer lesions. Occasionally, similar to lichen planus, there is acanthosis and thickening of the epidermis; however, similar to the patient in this report, the epidermis may be atrophic with flattening of the rete ridges. Hypergranulosis of the granular layer and sparse orthokeratosis overlying the epidermis may be observed [4,5,7,9,12,18,20].

Commonly, there is a band-like infiltrate of lymphocytes in the upper dermis; the pattern of inflammation is also referred to as lichenoid; alternatively, there may only be a sparse infiltrate of lymphocytes that are either perivascular or not surrounding the dermal vessels. The erythematous lesions of the reported patient also had numerous telangiectatic vessels in the upper dermis. Similar to the darker lesions of the patient described in this paper, the older lesions of lichen planus pigmentosus and lichen planus pigmentosus inversus characteristically demonstrate not only abundant melanin pigmentary incontinence but also features of post-inflammatory hyperpigmentation with numerous melanophages frequently being present in the upper dermis [4,5,7,9,12,18,20].

Potentially pathogenesis-related factors have been observed in lichen planus pigmentosus patients [4,8,9]. Sun exposure and ultraviolet radiation are also considered to be an exacerbating factor for the development of lichen planus pigmentosus; 40% (12 patients) of individuals with lichen planus pigmentosus in one retrospective study experienced photoaggravation of their lesions [9]. In a larger study of 124 lichen planus pigmentosus patients, 18% (22 patients) with lichen planus pigmentosus observed darkening of their pigmentation after exposure to sunlight [8]. 

Topical exposure to almond oil, amala oil for scalp and body massage, cold and cosmetic creams, and other toiletries (including aftershave lotions, deodorants, and perfumes) have been observed to be factors that may precipitate lichen planus pigmentosus and lichen planus pigmentosus inversus [5,8]. In addition to topical contact, consumption of mustard oil (which contains the photosensitizer allyl-thiocyanate) and nickel (in nickel-containing foods) have been observed to potentially promote these disorders [4,8]. Also, exposure to dyes, such as henna and paraphenyldiamine (in hair dye), has been identified as precipitating causes of these conditions [4,5,8].

Similar to lichen planus, lichen planus pigmentosus can be associated with hepatitis C [4,5]. Other conditions that have been associated with lichen planus pigmentosus and lichen planus pigmentosus inversus include endocrinopathies (diabetes mellitus), autoimmune conditions (hypothyroidism), and hyperlipidemia [4,5]. The lichen planus pigmentosus inversus patient in this report had not only diabetes mellitus of 10 years duration but also hyperlipidemia. He also had numerous axillary skin tags which could contribute to friction in this intertriginous area; however, an association of lichen planus pigmentosus inversus and acrochordons has not been previously reported.

In a study of 100 patients with lichen planus pigmentosus, associated diseases also included canities (premature graying or whitening of the scalp hair in 24 patients), hypothyroidism (22 patients), chronic urticaria (12 patients), tuberculosis (in five patients), hepatitis C (four patients), and vitiligo (one patient) [5]. A smaller study of 30 lichen planus pigmentosus patients observed one patient with each of the following conditions: cerebrovascular accident, chikungunya fever, diabetes mellitus, epilepsy, hypertension, hypothyroidism, and malaria [9]. Another study of 10 individuals with lichen planus pigmentosus inversus had either one (six patients), two (three patients), or three (one patient) associated medical conditions; these conditions included: high blood pressure (five patients), diabetes mellitus (three patients), mercury dental amalgams (two patients), cataracts (one patient), dyslipidemia (one patient), gastritis (one patient), hypothyroidism (one patient), and no associated medical condition (one patient) [20].

Recently additional potential etiologic factors of lichen planus pigmentosus inversus have been proposed: induction by radiotherapy, paraneoplastic-related, and associated with COVID-19 vaccination [10,13-17]. A 69-year-old woman developed lichen planus pigmentosus inversus localized only to the prior sites of radiotherapy on her left axillary region and left submammary area. She had received a total dose of 47.5 Gy of radiation treatment two years earlier for left breast carcinoma [14].

Lichen planus pigmentosus inversus and lichen planus pigmentosus have been described in oncology patients [10,13-15]. A 69-year-old woman with carcinoma of the left breast developed lichen planus pigmentosus inversus restricted to the radiotherapy treatment sites two years after receiving treatment [14]. A 76-year-old man developed lichen planus pigmentosus inversus affecting his bilateral axillae and inguinal area three years after being treated with a unilateral mastectomy for breast cancer [13].

Paraneoplastic lichen planus pigmentosus inversus was observed in an 81-year-old man with two cancers. He initially developed lichen planus pigmentosus inversus when his symptoms of colorectal cancer began six months earlier. One month after his colorectal cancer was treated surgically, laryngeal carcinoma was diagnosed; the cancer of the larynx was surgically excised, and he received radiotherapy. His lichen planus pigmentosus inversus lesions persisted following treatment of both cancers, and he was lost to follow-up [15]. 

Paraneoplastic lichen planus pigmentosus was observed in a 50-year-old man. He concurrently developed lichen planus pigmentosus and acrokeratosis of Bazex; bilateral cervical adenopathy associated with metastatic poorly differentiated epidermoid carcinoma was discovered. A primary neoplasm was not detected; however, both cutaneous conditions disappeared after treatment of the neoplasia with radiotherapy and chemotherapy [10]. The researchers also identified two additional patients who had a lichenoid dermatosis similar to their patient, Bazex’s acrokeratosis, and either a laryngeal or pharyngeal carcinoma [10].

Lichen planus pigmentosus inversus has been reported in three individuals following the administration of the COVID-19 vaccine. The first patient was a 64-year-old woman who initially developed lichen planus pigmentosus inversus two weeks after her inoculation of the first dose and had clinical worsening after the second dose of the same vaccine. The second patient was a 52-year-old man who noted that his skin lesions of lichen planus pigmentosus inversus began one week after the third dose of the vaccine, and dermatosis-related nail changes appeared two weeks after the presentation of his skin lesions. The third patient was a 71-year-old man whose lichen planus pigmentosus inversus skin lesions appeared two weeks after receiving the second dose of the vaccine [16,17].

Several investigators have suggested that the pathogenesis of lichen planus pigmentosus is similar to that of lichen planus. Most of the studies of lichen planus pigmentosus pathogenesis show a predominance of cluster of differentiation-8 (CD8)-positive T-lymphocytes over cluster of differentiation 4 (CD4)-positive T-lymphocytes. However, one group of investigators noted early predominance of both CD4-positive T-lymphocytes and CD8-positive T-lymphocytes; the researchers concluded that the early increase in CD4-positive T-lymphocytes might warrant prompt treatment that is targeted to arrest the disease’s autoimmune course and to prevent inflammation persistence [9].

Recent evaluation of immune responses has shown that the expression of T-helper 17 (Th17)-related genes, interferon-gamma, and forkhead box P3 (FOXp3) in lichen planus pigmentosus is significantly reduced as compared to lichen planus; hence, this raises the possibility that the pathogenesis of these two diseases may differ. In addition, a pilot study evaluating lichen planus pigmentosus inversus using a dermal biomarker patch and whole transcriptome analysis not only confirms interferon signaling and T-cell activation in lichen planus pigmentosus inversus but also suggests that the expression profile of lichen planus pigmentosus inversus is distinct from that of lichen planus. Specifically, there is significant upregulation of the genes responsible for not only an intergenic splice variant of the primary pigmentation determining receptor in humans but also the dysregulation of genes essential for ceramide synthesis and construction of the cornified envelope when biopsies evaluated from lichen planus pigmentosus inversus lesions are compared to biopsies from those of lichen planus lesions [18].

The pigmentary manifestations of lichen planus pigmentosus and lichen planus pigmentosus inversus are primarily of cosmetic concern; for many of the patients, the dyschromia is refractory to treatment. Indeed, there are individual reports of successful management of these conditions. However, most of the therapeutic interventions that have been used for patients with these disorders have not been uniformly effective [3,4,7,8].

Removal of causes that have been associated with the etiology of lichen planus pigmentosus and lichen planus pigmentosus inversus has been suggested. Since sun exposure may be a risk factor for lichen planus pigmentosus, therapeutic interventions include not only avoiding phototherapy because there is an increased risk of hyperpigmentation but also initiating photoprotection by regularly applying sunscreen to the affected area. Some investigators have suggested that it may be helpful to treat condition-related comorbidities such as autoimmune disorders, dyslipidemia, hepatitis C, and hypothyroidism [1,3,4].

For lichen planus pigmentosus inversus, avoidance of potential triggers should be initiated. Fragrances and deodorants should be eliminated. In addition, individuals should stop wearing tight clothing that causes friction to the adjacent skin [3,4,11]. Two Japanese patients had disappearance of the lesions after discontinuation of wearing tight underclothes [19].

Several researchers observed that high and medium potency topical corticosteroids had minimal to no improvement [3,4,11]. The topical corticosteroid included clobetasol propionate [3,4,11]. It also included betamethasone in 10 patients [20].

In addition, when the higher potency topical corticosteroids were used in intertriginous areas, skin atrophy would result [3,4,11,20]. Similarly, other investigators also found that using a low potency topical corticosteroid cream in a 50-year-old woman with lichen planus pigmentosus inversus involving skin cleavage lines over the axillae and groin provided little improvement after three months [19]. Also, oral prednisone did not result in improvement of the lesions [11].

Monotherapy with calcineurin inhibitors, such as 0.03% and 0.1% tacrolimus ointment, has been reported to be efficacious [3,4,7,13]. In a 76-year-old man with bilateral axillary and inguinal lesions of lichen planus pigmentosus inversus, once daily topical application of 0.1% tacrolimus over a four-month period resulted in the intertriginous lesions diminishing their pruritus, reducing their size, and becoming lighter and flatter. The investigators considered the treatment with the topical calcineurin inhibitor to be safe, effective, and simple [13].

A 71-year-old man who developed lichen planus pigmentosus inversus two weeks following the second dose of COVID-19 vaccine had partial clearance of the lesions after three months of treatment using once daily topical clobetasol propionate 0.05% and tacrolimus 0.1% [16]. Also, in another study, 53.8% of the patients found twice daily application of tacrolimus ointment for six to 12 weeks to be an effective treatment; however, other investigators did not observe any significant response after four weeks of treatment using clobetasol propionate 0.05% and tacrolimus 0.1% twice daily in four patients [3,19]. In addition, the lesions of a patient with lichen planus pigmentosus inversus, who was treated twice daily with topical tacrolimus, had no change after four weeks [19]. 

In addition to oral corticosteroids, other reports have described a poor response to other systemic agents [11]. A poor response to chloroquine monotherapy has been observed [1]. In addition, only moderate improvement was observed in a 59-year-old woman with lichen planus pigmentosus who received six months of combination therapy including hydroxychloroquine 200 mg twice daily, topical tacrolimus 0.1% ointment, and sunscreen [6].

Systemic dapsone and topical tacrolimus along with photoprotection have been reported to be effective in controlling the progression of lichen planus pigmentosus [7]. In a study of five lichen planus pigmentosus patients, a successful response to this combination therapy was observed. The patients were treated with 100 mg daily of dapsone for four months and applied 0.1% tacrolimus twice daily [4].

Oral retinoids have been used as monotherapy and in combination therapy to treat lichen planus pigmentosus and lichen planus pigmentosus inversus. In a study of 32 lichen planus pigmentosus patients, 27 of the patients completed six months of daily treatment with 20 mg of isotretinoin. In four to six weeks, stabilization of the disease was achieved, and within three months, decreased pigmentation was observed. Improvement was assessed as good (seven patients), moderate (15 patients), mild (two patients), and none (three patients) [4].

Acitretin, 10 mg twice daily, was used in three women with lichen planus pigmentosus inversus in combination with a Huayu decoction twice daily, which is a combination of Chinese herbs. After three months of treatment, the hyperpigmented macules and patches became noticeably lighter [19]. Another group of investigators also achieved good results in six lichen planus pigmentosus inversus patients with Huayu decoction monotherapy [19].

Attempts to treat the dyschromia have included numerous topical depigmenting agents including hydroquinone 4%, kojic acid, and modified Kligman’s formula [4]. The depigmenting agents were usually used as an adjuvant therapy along with other topical or systemic treatments [4]. In addition, lasers that target skin pigmentation such as the Q-switched laser (1064 nanometer neodymium doped-yttrium aluminum garnet) have also been used [3,4,7].

A 50-year-old man patient who had paraneoplastic lichen planus pigmentosus and concurrent acrokeratosis of Bazex presenting with bilateral adenopathy of his cervical lymph nodes secondary to a poorly differentiated metastatic epidermoid carcinoma of unknown primary. His cancer was successfully treated with radiotherapy and chemotherapy (cisplatin and fluorouracil) treatment; five years later, he was still cancer-free. His acrokeratosis disappeared within three months after completing treatment. In contrast, his lichen planus pigmentosus-associated pigmentation persisted and was resistant to topical treatment with hydroquinone. However, a follow-up one year later demonstrated that the lichen planus pigmentosus-related pigmentation, which had initially appeared after a short period of sun exposure and had occurred in both sun-exposed and nonexposed areas, had regressed. During the five subsequent sunny seasons, neither his photosensitivity nor the lichen planus pigmentosus recurred [10].

## Conclusions

Lichen planus pigmentosus inversus is a rare variant of lichen planus pigmentosus. Lesions of lichen planus pigmentosus inversus appear as hyperpigmented patches or plaques in intertriginous areas; when affecting the axilla, the dermatosis has been described as a PLAID. A Hispanic man is described who developed lichen planus pigmentosus inversus that presented with a PLAID. His dermatosis-related skin lesion had two patterns of presentation. All the lesions in his left axilla were scaly hyperpigmented plaques; some of the slightly scaly plaques in his right axilla had central erythema with surrounding hyperpigmentation. Skin biopsies of the red plaques and dark plaques showed similar pathologic changes. There was orthokeratosis overlying an atrophic epidermis which had flattening of the rete ridges. A lichenoid infiltrate of lymphocytes was present in a band-like distribution in the papillary dermis. The red plaque had several telangiectatic vessels and sparse melanophages in the upper dermis. In contrast, the darker plaque had focal vacuolar alteration of the cells in the basal layer of the epidermis; in the upper dermis, there was not only edema but also numerous melanophages. Correlation of the clinical presentation and pathologic changes on lesional skin biopsies established the diagnosis of lichen planus pigmentosus inversus. The clinical differential diagnosis of lichen planus pigmentosus inversus includes inherited disorders, primary cutaneous dermatoses, acquired dyschromias, and reactions to topical or systemic medications. Almond oil, amala oil, cold and cosmetic creams, henna, friction in intertriginous areas, mustard oil, nickel, and paraphenyldiamine can exacerbate lichen planus pigmentosus inversus. Diabetes mellitus, hyperlipidemia, and hypothyroidism can potentially be associated with lichen planus pigmentosus inversus. Lichen planus pigmentosus inversus can be refractory to treatment. Management of lichen planus pigmentosus inversus may include not only removing tight-fitting clothing to prevent friction to the adjacent skin but also topical calcineurin inhibitors such as tacrolimus. In conclusion, a PLAID can be the presenting clinical manifestation of either inverse lichen planus or lichen planus pigmentosus inversus; in contrast to lichen planus, lichen planus pigmentosus inversus rarely benefits from topical corticosteroids and may show some improvement with topical tacrolimus.
